# Failure of motor function—A Developmental Embodiment Research perspective on the systemic effects of stress

**DOI:** 10.3389/fnhum.2023.1083200

**Published:** 2023-02-16

**Authors:** Melanie Krüger, Vanessa Lux

**Affiliations:** ^1^Institute of Sports Science, Faculty of Humanities, Leibniz University Hannover, Hannover, Germany; ^2^Department of Genetic Psychology, Ruhr University Bochum, Bochum, Germany

**Keywords:** movement coordination, embodied cognition, motor control, dual tasking, stress response, lifespan perspective, psychobiological development

## Abstract

Humans are capable to skillfully perform a huge variety of complex movements seemingly effortless and to flexibly adjust movement execution to ever-changing environmental conditions, often without apparent differences in the movement outcome. This impressive ability has sparked scientific interest in the mechanisms underlying movement execution for decades. In this perspective article, we argue that investigating the processes and mechanisms leading to failure of motor functions is a fruitful approach to advance the field of human motor neuroscience and beyond. The study of failure of motor function in specific populations (patient groups, skilled experts) has already provided tremendous insight in the systemic characteristics and multi-level functional dependencies of movement execution. However, particularly the transient failure of function in everyday motor actions remains a blind spot. Coming from the perspective of Developmental Embodiment Research, we argue that the integration of a developmental embodiment and lifespan perspective with existing systemic and multi-level methodological approaches of failure of function analyses provides an integrative, interdisciplinary framework, which will allow us to overcome this shortcoming. We further suggest that stress-induced failure of motor function situations might represent a promising starting point for this endeavor. Identifying the involved cross-level functional dependencies of acute and chronic stress on transient and persistent motor functioning would further advance our knowledge on the mechanisms underlying movement execution, and would allow to identify targets for intervention and prevention across the whole spectrum of motor function and failure.

## 1 Introduction

Humans have the capability to skillfully perform complex movements seemingly effortless and to flexibly adjust movement execution to ever-changing environmental conditions, often without apparent differences in the movement outcome. This impressive ability has caught scientific interest about the underlying mechanisms enabling as well as controlling skillful movement execution for decades. However, in a recent article, Levin and Piscitelli (2022) state that a unifying theory of motor control is still missing. Simultaneously, they highlight an increasing awareness about *“how knowledge of normal control processes may inform the understanding of disordered movement production to effect better functional outcomes”* (Levin and Piscitelli, 2022, p. 500). Within this article, we will argue that also the opposite—learning from failure i.e., investigating the processes and mechanisms leading to failure of motor functioning to gain understanding about “normal motor control processes”—might be a fruitful approach to advance scientific knowledge and insight in the field of human motor neuroscience and beyond.

We will do this from the perspective of Developmental Embodiment Research. The approach emphasizes the multi-level and dynamic-interactionist character of the human developmental system and employs an interdisciplinary and lifespan perspective on embodiment processes (see Lux et al., 2021). One proposition of Developmental Embodiment Research is to study cross-level dependencies and their developmental dynamics to identify mechanisms and pathways of bodily manifestations related to the emergence and maintenance of function and its failure. Most specifically, the goal is to describe nodes of cross-level interactions at which experiences are incorporated via translational processes between the involved functional levels. We will argue that this perspective is specifically suited to study transient failure in everyday motor function, and propose that stress, with its acute and chronic impact on various domains of system function, is a good starting point to capture the systemic and multi-level dependencies of the mechanisms underlying motor function and failure.

## 2 Failure of function—research

Failure of function approaches contributed to groundbreaking findings within different fields of research. Nowadays, failure of function investigations span from business studies (Edmondson, 2004, 2011; Cannon and Edmondson, 2005; Desai et al., 2017) and architecture (Adam et al., 2018; Buchanan, 2019), to engineering (Stone et al., 2005), safety studies (Rasmussen, 1997; Constantinides, 2013), and computer sciences (Schroeder and Gibson, 2009; Rambikur et al., 2017). In the bio- and life sciences, the potential of failure of function approaches has been successfully exploited, for example, by lesion studies in neuroscience (Damasio and Damasio, 1989; Vaidya et al., 2019) and psychology (Keysers et al., 2018; Mazzi and Savazzi, 2019), in prosthetics studies (Gratton et al., 2002; Williams and Chawla, 2014; Sturma et al., 2021) and by the use of knock-out models in genetics (Nelson, 1997, 2015; Deutscher et al., 2008).

Across these disciplines, we can differentiate two types of failure of function analytical approaches: (1) *Retrospective and* *prospective failure analyses* as conducted in engineering, computer sciences, safety studies, and research on learning organizations (ref. see above). In these cases, based on efficient data collection strategies, the analyses are conducted to identify the (potential) failure as well as the range of conditions leading to it, and (2) *Analyses of artificially induced or known failures* e.g., in genetic knock-out models or lesion studies, respectively, to study further systemic consequences. In some areas, both types of analyses are combined: in prosthetics studies, for example, the prosthesis is expected to compensate functional failure due to the bodily loss and, in addition, functional failure of the prosthesis’ material needs to be monitored and forecasted to avoid future harm (as in the case of the prospective lifespan of a pacemaker).

### 2.1 Failure of function in complex systems

From the perspective of Developmental Embodiment Research, failure of function is grounded in the loss or interference of necessary (physiological, embodied) preconditions, involving multiple system levels. Importantly, in complex systems, such as the human motor system, failure is often only partial and gradually defined, due to the involvement of multiple interacting pathways and buffering mechanisms across system levels. To investigate the cross-level dependencies underlying failure of function in complex systems, it is therefore fruitful to understand function and failure as points along a continuum, and with it, to acknowledge short- and long-term functional variability.

In addition, one core feature of failure of function approaches is their multi-methods use, combining a broad range of data with different data modalities and sampling contexts, ranging from bio-/chemical analysis, visual inspection, material tests, simulation studies, behavioral analysis, participatory observation, and qualitative interviews (Booker et al., 2022). Also, psychological models of perception, decision making, and attitudes have been widely applied for failure analysis (e.g., human-robot interactions: Honig and Oron-Gilad, 2018, 2021). This multi-method approach makes failure of function research particularly suitable for the investigation of motor functioning, an interdisciplinary field of research from the ground.

### 2.2 Failure of motor function

In the field of human movement science, independent of whether approaching it from a neuroscientific, psychological or sports science perspective, research on failure of motor functions typically focusses on two observations. On the one hand, *persistent*, potentially progressive impairments of *everyday motor functions* due to neurological injury or pathology, as for example (hemi-) paresis after stroke or parkinsonian motor disturbances, with the aim of developing rehabilitation interventions (Morris and Iansek, 1996; Cirstea and Levin, 2000; Iansek and Danoudis, 2016). Failure of motor function in these patient populations is rather general, situation-independent and with limited functional improvements due to training (for a recent review on the effectiveness of proprioceptive training, see Aman et al., 2015). Here, failure of function research provided evidence for the parallelism of recovery and compensatory mechanisms in the (partial) restoration of motor functioning, involving different system levels to become behaviorally effective.

On the other hand, research has focused on *transient* failure phenomena of *skilled motor behavior* in different groups of motor experts e.g., yips in golf and tennis players (Clarke et al., 2015; Papineau, 2015), focal dystonia in professional musicians (Altenmüller and Jabusch, 2009, 2010) or choking (Baumeister, 1984; DeCaro et al., 2011; Mesagno and Beckmann, 2017). The latter case has attracted scientific interest particularly in the field of sports psychological research in elite level sports, where motor functioning is pushed to the limits and slightest changes in system functioning might lead to significant performance decline. Besides neurophysiological correlates and motor manifestations, also different psychological factors seem to be related to the occurrence of these transient failure phenomena, facilitated by the perception of pressure in performance situations (Altenmüller and Jabusch, 2009; Klaempl et al., 2020). Consequently, recent research emphasizes the use of multi-dimensional theoretical conceptualizations and corresponding research designs, as well as multi-dimensional coping and intervention strategies to treat transient failure of motor function phenomena (Clarke et al., 2015).

### 2.3 The blind sport in failure of motor function—research

In sum, research on persistent failure of everyday motor functions in different patient populations provided insights into compensatory mechanisms contributing to the (partial) restoring of motor functions, while research on transient failure of skilled motor behavior in expert populations provided insights into the multi-level etiology and character of failure. However, *transient decline and failure* of motor functioning can also be observed *in everyday motor actions* e.g., in the case of stuttering, transient gait disturbances while walking, or while dual-tasking. The transience of its occurrence might be indicative for the involvement of interacting and mediating pathways, as well as buffering mechanisms across system levels to restore and stabilize motor functioning. One mechanism assumed to stabilize motor performance is the exploitation of redundancy within and across multiple levels of the human motor system (Todorov and Jordan, 2002; Davids et al., 2003; Latash et al., 2007). In this context, the analysis of variability in movement execution, e.g., at the level of muscle activity or inter-joint coordination, provided evidence for the assumption that synergistic coordination allows to stabilize motor functioning by simultaneously granting necessary flexibility in movement execution.

Furthermore, motor functions are subject to developmental changes across the lifespan (Haywood and Getchell, 2021). Consequently, failure of motor functions might be, at least to some extent, governed by *developmental changes* at different system levels, *potentially linking transient to persistent failure*. While some of the mechanisms underlying transient and persistent failure might be specific to the situational aspects under which failure occurs, others might overlap. Here, stress-induced failure of motor function-situations represent a promising starting point for further investigations, as stress is known to have transient but also persistent effects on systemic functioning in various domains. Further, stress is not only known to affect functioning at different system levels, but also to originate from different levels (e.g., physiological and psychological) and to act at different time scales, namely acutely vs. chronically.

## 3 Systemic origins and effects of stress

The stress system is a powerful mediator and response system between external inputs and the body and mind. One of its core functions is to mobilize the bodies energy resources and protective systems in light of a vital threat (Segerstrom, 2007; Chrousos, 2009). For this purpose, the stress system, roughly speaking, comprises of two response cycles, a short-term response involving the secretion of catecholamines, including adrenalin and noradrenalin, and a long-term response based on the hypothalamic-pituitary-adrenal (HPA) axis involving the secretion of glucocorticoids, specifically cortisol in humans (Chrousos, 2009). Both response cycles are activated during an acute stressful situation and serve the purpose to maintain function under these circumstances. In healthy individuals, the feedback mechanisms in both response cycles lead to readaptation to the original set-points after the stressful situation is overcome. However, when a stressful situation is either extreme, prolonged, or recurrent, it seems that the readaptation of the long-term response cycle, involving the HPA axis, shifts towards a different set-point, resulting in a less pronounced response to future stressors and even hypocortisolism (Heim et al., 2000; Fries et al., 2005). From a systemic perspective, this re-regulation of set-points makes sense, as enduring high levels of cortisol over a longer period may be harmful to several bodily functions including the metabolic systems, the immune system, and even basic cellular functions (Fuchs and Flügge, 2004; Fries et al., 2005; Chrousos, 2009; Cohen et al., 2012). Both, high levels of cortisol as well as a dampened cortisol reaction in response to acute stress have been implicated with the long-term impact of chronic stress on general and mental health (Heim et al., 2000). Thus, the stress system can induce protective as well as harmful changes in a systemic and highly individual manner (Chrousos, 2009; Claessens et al., 2011).

### 3.1 Developmental dimensions of stress and stress-induced failure of function

From a developmental perspective, we can characterize the stress system as a system that effectively helps to maintain and stabilize function when function is in crisis. Being part of the body’s guard rails in maintaining homeostasis, it affects body and mind at different functional levels all the way down to the underlying cellular and molecular processes. Development is a key strategy for organisms to stabilize and maintain function, sometimes even by giving rise to new functions (Baltes, 1987). The stress system contributes to ontogenetic development in different ways, for example, by influencing sensitive periods or by facilitating and accelerating developmental processes. It potentially serves as mobilizing factor, opening developmental windows for systemic adaptation (for the discussion of this impact on epigenetic developmental mechanisms see Lux, 2018).

One example for the developmental impact of stress is the role of glucocorticoids in organ development. Glucocorticoids accelerate organ maturation during embryonic development (Ballard and Ballard, 1995; Grier and Halliday, 2004). The maturation processes are present down to the cellular level, with developmental changes being reflected in changes of gene transcription and protein synthesis. Newer studies also show functional relevant modifications of DNA methylation (Crudo et al., 2012; Khulan et al., 2016). Another example is the effect of stress on brain and cognitive development. The detrimental role of high levels of prenatal stress on brain development is well established, affecting memory and executive functions (Charil et al., 2010; Lautarescu et al., 2020). Epidemiological data and longitudinal clinical studies also indicate that increased levels of stress, either in form of stressful life events or as perceived stress in general, are associated with earlier onset of cognitive decline and higher risk for neurodegenerative diseases in old age (see, for example, Peavy et al., 2012; Scott et al., 2015; Koyanagi et al., 2019).

Hence, stress-induced systemic adaptations may result in functional improvement or stabilization of function through short-term mobilization of energy resources or developmental acceleration, but may also be the source of functional failure or gradual functional decline, acutely and in the long run, and specifically in the case of repeated or prolonged stress exposure. Furthermore, biological embedding of stress-induced functional changes at different system levels can potentially result in long-term vulnerabilities, which could then lead to failure of function of specific systems or at specific system levels later in life. Such life-time vulnerabilities also likely contribute to individual differences in stress sensitivity over the lifespan.

While stress, as these examples show, heavily impacts not only the current state but also the development and the future state of body and mind, the perception of a psychosocial stressor as stressful also depends on previous stress experiences (Epel et al., 2018). For example, extreme or even traumatic as well as recurrent experiences of stressful situations could lower the threshold for a situation to be perceived as stressful and the health impact of minor stressors, as seen in the effects of early childhood adversity (Smith and Pollak, 2020), war crime trauma (Miller and Rasmussen, 2010), and different types of cumulative stress exposure due to bullying (Östberg et al., 2018), racial and gender discrimination (Perry et al., 2013), and daily hassles (DeLongis et al., 1982). In contrast, previously experienced stressful life events, when successfully overcome, could also provide resources against the impact of future similar stressors due to adapted coping strategies. Overall, a developmental and lifespan perspective is necessary to fully capture the impact of stress and stress-induced failure of function.

### 3.2 The influence of stress on motor function

Both, acute and chronic stress are known to have a significant impact on motor functioning. Acute stress has been related not only to beneficial but also detrimental changes in transient motor functioning, such as fine motor control, reaction time, and movement speed. This relationship is often described as an “inverted U-shape function” (Welford, 1973). In a comprehensive review, Metz (2007) emphasizes that a prolonged experience of a stressor may increase disease vulnerability. Specifically, Metz (2007) suggests the potential central relevance of chronic stress for the development and progression of neurodegenerative diseases, with implications for postural control and locomotion. Importantly, the stress-performance relationship in motor performance seems to be contextual and complex, requiring multidimensional theoretical and methodological approaches (Jones and Hardy, 1989; Van Gemmert and Van Galen, 1997; Metz, 2007). As such, multiple stressors were investigated on their influence on motor functioning in healthy as well as patient populations. This included psychological stressors using emotional stimuli (Blakemore et al., 2018), social stressors using a validated stress test (Apazoglou et al., 2017), physical, and most often cognitive stressors (Metz, 2007). Van Gemmert and Van Galen (1997), for example, investigated the differential effect of physical and cognitive stress on motor performance, with empirical evidence suggesting a stronger influence of cognitive stress, induced through cognitive-motor dual-task demands, on motor performance.

Cognitive-motor dual-tasking is an everyday phenomenon, observable e.g., when dealing with the phone while walking or when participating in a conversation while performing household tasks. Bridenbaugh and Kressig (2011) term the cognitive-motor dual-task paradigm a “cognitive stress resistance model” (p. 260) with stress effects on transient motor functioning suggested to increase with increasing cognitive load. Different theories try to explain dual-task performance decrements and the underlying neurophysiological, cognitive, and motor processes, with the most prominent set of theories ascribing their occurrence to resource limitations of a central controller when having to parallelly process multiple tasks demands which overlap in time or domain (for an overview, see e.g., Heuer, 1996). Importantly, failure of motor functions under dual-task demands are usually only partial, quantified as “dual task costs”, and found to increase with increasing age (Schaefer, 2014). Further, failure of motor functions under dual-task demands are inherently transient, as a strategic separation or the prioritization of the dual-task demands results in the immediate restoration or lower performance degradation, respectively (Lundin-Olsson et al., 1997; Schaefer, 2014).

In sum, acute and chronic stress have both been found and suggested to affect motor functioning directly but also indirectly in everyday motor task, due to complex links between changes in hormonal, (neuro-)physiological, psychological, cognitive, and motor functions. However, whether a developmental trajectory from transient to persistent failure exists in the context of stress-induced failure of everyday motor function remains to be investigated. Here, a combination of measures, including (neuro-)physiological, cognitive, biomechanical, and kinematic measures, is most desirable to register functional changes at different system levels. Furthermore, previous lifetime and chronic stress need to be assessed to consider lifespan effects of stress on the susceptibility to failure of motor function. With its multi-methods approach to identify cross-level functional dependencies and its explicit consideration of the system’s complexity, Developmental Embodiment Research provides a powerful framework to study these questions.

## 4 Future perspectives

Several avenues for future research on failure of motor function derive from a Developmental Embodiment Research perspective on systemic stress effects: First, by *acknowledging the developmental dimensions of stress-effects* the impact of previous stress experiences on situation-dependent transient variability in motor functioning, but also the progression to persistent failure of function gets into focus. Specifically, investigating transient failure from a developmental perspective could reveal nodes of cross-level dependencies and mechanisms of translation between system levels, due to which transient failure becomes persistent and including those that participate in the transition from performance-enhancing to detrimental effects of stress. These nodes, and the way they arise and are maintained during development, might constitute targets for preventive and rehabilitative interventions in the future. Moreover, identification of *developmental effects of stress on motor function in everyday activities and its systemic embeddedness* then also reversely allows to infer function from failure.

Accordingly, methodological approaches are necessary which enable to *identify changes in underlying motor functioning in the absence of overt changes in movement performance*. Here, focusing on synergistic movement coordination by investigating changes in the structure of movement variability provides a powerful approach, already proven to identify compensatory movement strategies in patients and older adults (e.g., Latash et al., 2010; Krüger et al., 2013) and shown to be affected by stress (Gray, 2011). Further, to *identify translational mechanisms mediating stress-effects between system levels*, multi-factorial, longitudinal research designs are needed, preferably combining behavioral and computational approaches.

## 5 Conclusion

While failure of motor function has received considerable scientific attention with regard to the persistent failure of everyday motor actions in patient populations, or the transient failure of skilled movement execution in expert populations, the transient failure of function in everyday motor actions remains a blind spot in human motor neuroscience and related fields of research. We suggest that investigating stress-induced failure of motor function e.g., in dual-task settings, might represent a promising starting point to overcome this shortcoming. In that regard, a Developmental Embodiment Research perspective provides a powerful framework, as it integrates developmental dimensions of acute and chronic stress on motor functioning with multi-method approaches involving different system levels for the fine-grained analysis of motor function. This approach would further advance our knowledge on the mechanisms underlying movement execution, and would allow to identify targets for prevention and intervention across the whole spectrum of motor function and failure.

## Data availability statement

The original contributions presented in the study are included in the article, further inquiries can be directed to the corresponding author.

## Author contributions

MK and VL: manuscript preparation in every step i.e., conception, drafting, and revision. Both authors contributed equally to the article and approved the submitted version.
